# Supplementary figures and images for: Archaea produce peptidoglycan hydrolases that kill bacteria
Source: PLoS Biol. 2025 Aug 14;23(8):e3003235. doi: 10.1371/journal.pbio.3003235 (PMC12352652; doi:10.1371/journal.pbio.3003235)

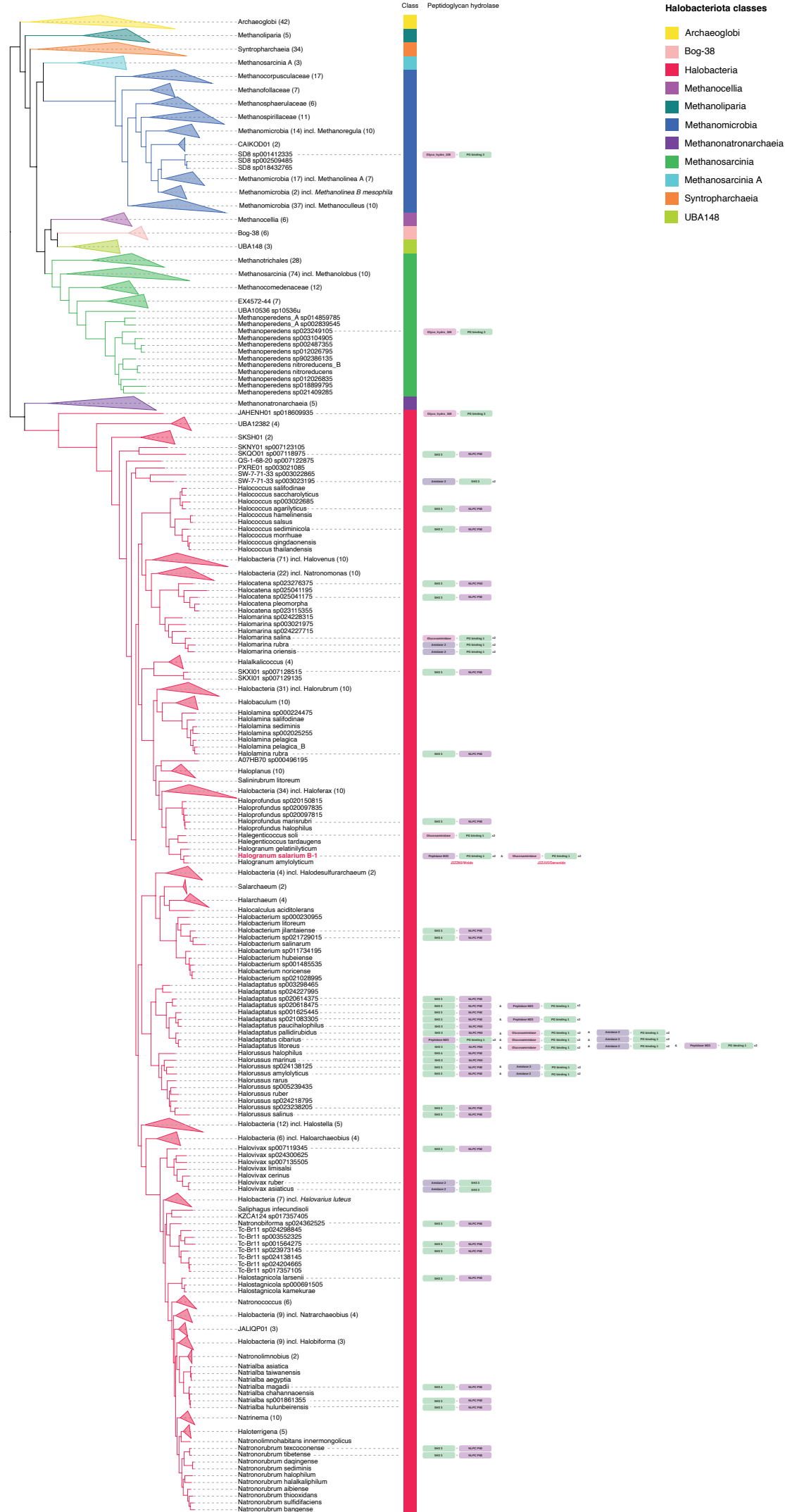

Supplement: S1 Fig — The tree shown is based on the GTDB archaeal tree but pruned to only contain Halobacteriota species present in our database. Tree scale: substitutions per site. Code and data are available at zenodo.org/records/15534318 (“notebook/figureS1.ipynb” and “data/figureS1/”, respectively). (PDF) [file pbio.3003235.s001.pdf]

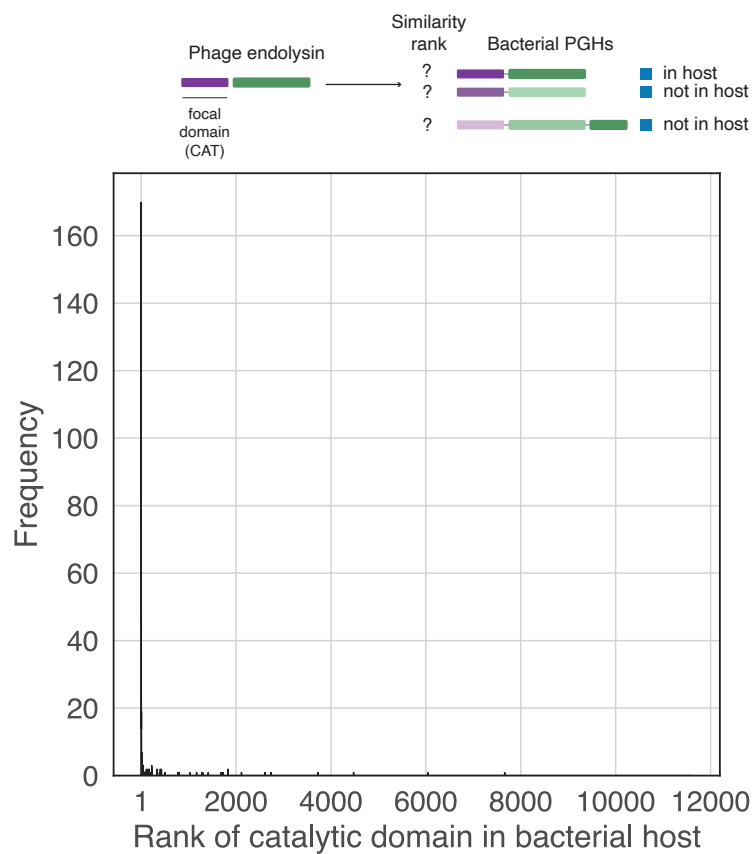

Supplement: S2 Fig — This figure corresponds to Fig 3B but for the catalytic (CAT) instead of the cell wall binding domain. Code and data are available at zenodo.org/records/15534318 (“notebook/figure3.ipynb” and “data/figure3/”, respectively). (PDF) [file pbio.3003235.s002.pdf]

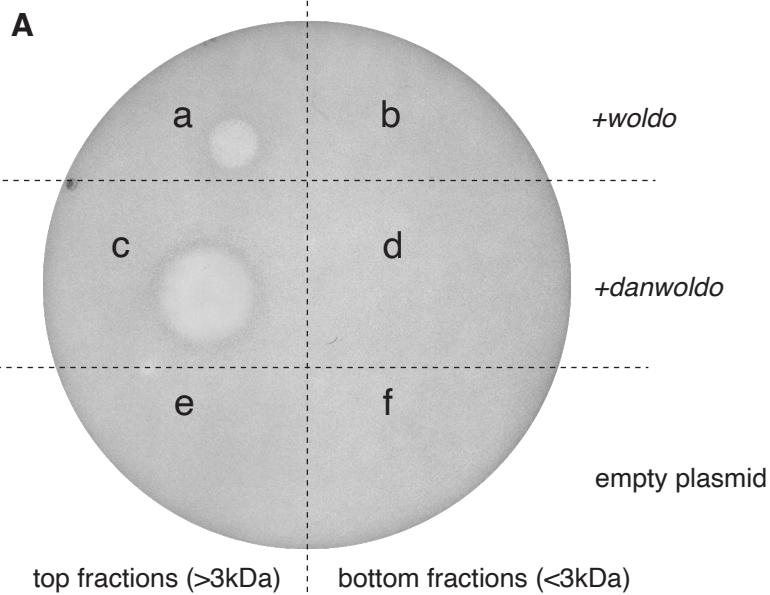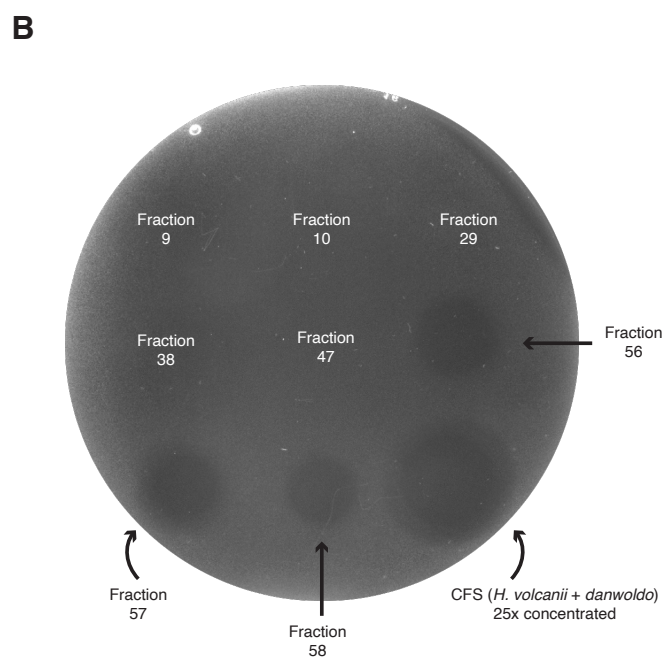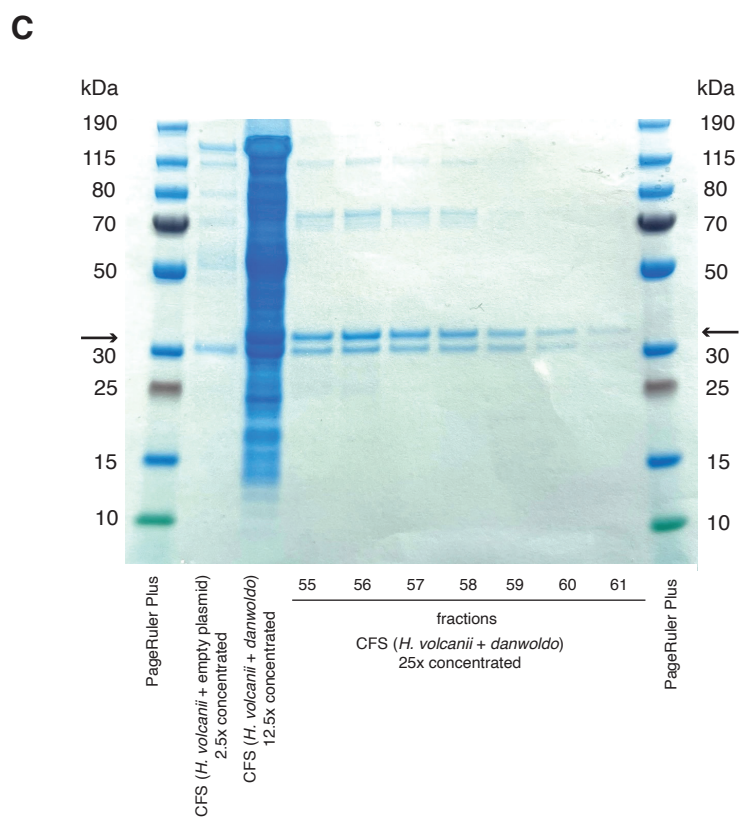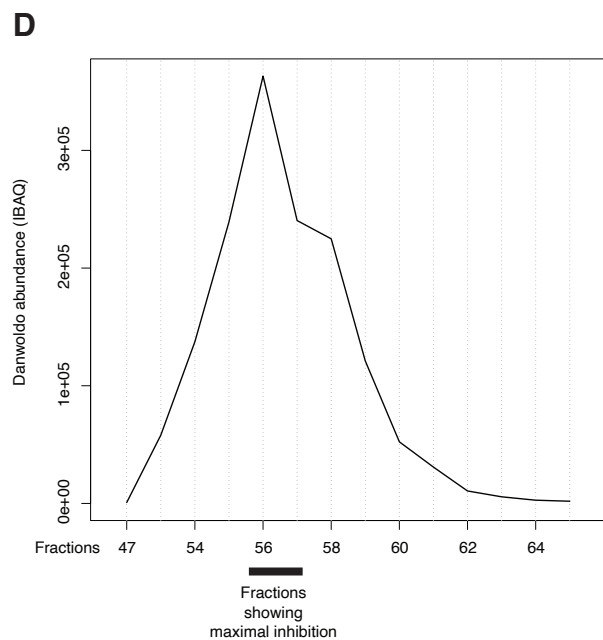

Supplement: S3 Fig — A. Comparing the inhibitory effects of Woldo and Danwoldo on the same plate. The following supernatants were spotted onto a lawn of H. halodurans, a,c,e: top fraction (>3 kDa) of supernatant from H. volcanii (a) expressing Woldo, (c) expressing Danwoldo, (e) with an empty plasmid; b,d, f: bottom fraction (<3 kDa) of supernatant from H. volcanii (b) expressing Woldo, (d) expressing Danwoldo, (f) with an empty plasmid. B. Lawn of H. halodurans exposed to the cell-free supernatant (CFS) of danwoldo-expressing H. volcanii, and defined fractions of that supernatant (see Methods). C. Coomassie-stained SDS-PAGE gel highlighting the protein contents of peak killing fractions (56/57) and neighboring fractions. The expected size of Danwoldo is indicated by arrows. D. Protein abundance profile (based on IBAQ measurements from quantitative proteomics, see Methods) of Danwoldo across killing and neighboring fractions. Code and data are available at zenodo.org/records/15534318 (“notebook/figureS3.R” and “data/figureS3”), respectively. (PDF) [file pbio.3003235.s003.pdf]

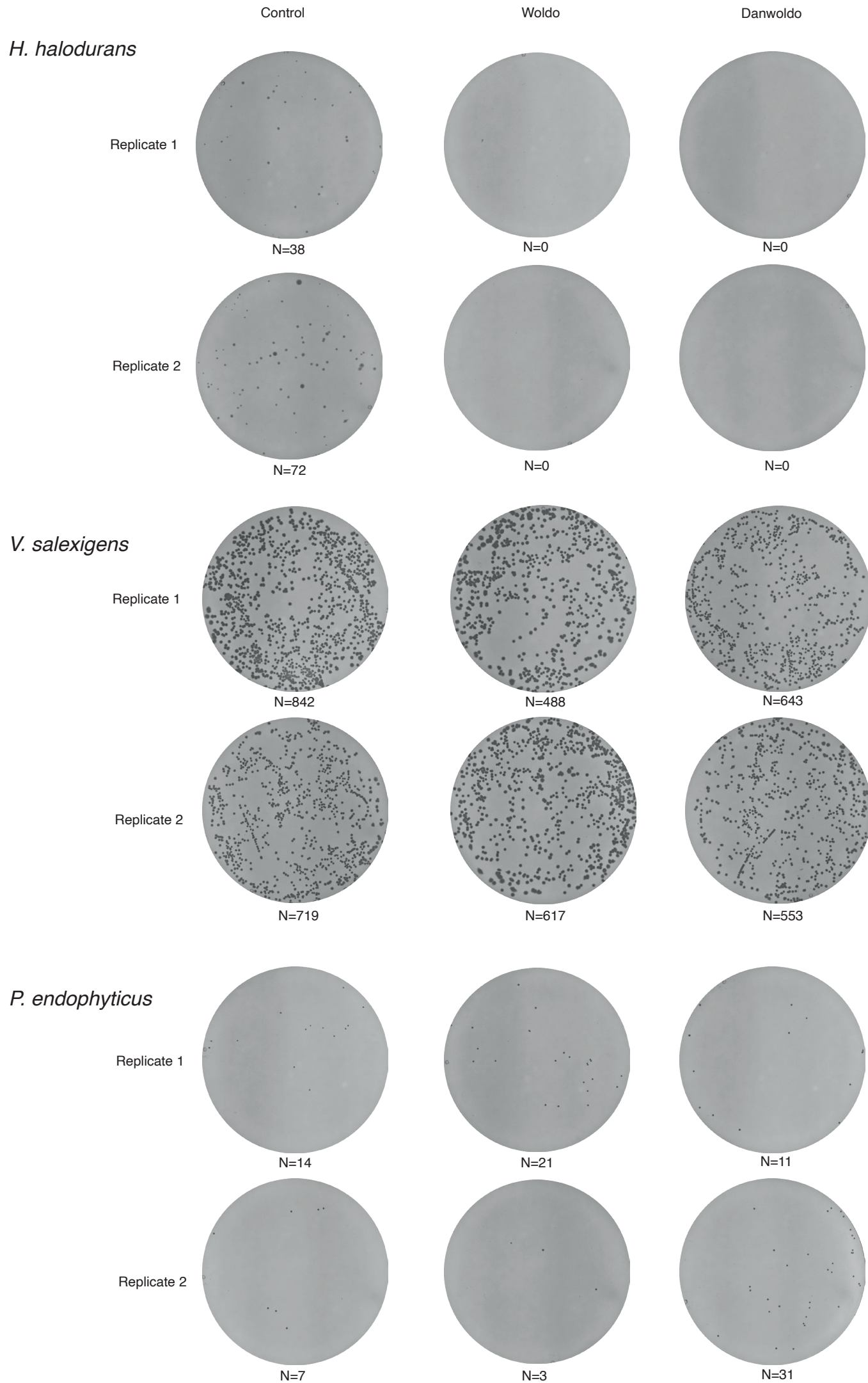

Supplement: S4 Fig — (PDF) [file pbio.3003235.s004.pdf]
