# Supplementary material for: Archaea produce peptidoglycan hydrolases that kill bacteria
Source: PLoS Biol. 2025 Aug 14;23(8):e3003235. doi: 10.1371/journal.pbio.3003235 (PMC12352652; doi:10.1371/journal.pbio.3003235)
Supplement: Attachment — Submitted filename: response_to_reviewers2.pdf [file pbio.3003235.s018.pdf]

Point-by-point response to reviewers

General comments:

- We thank the four reviewers for their constructive feedback on our manuscript. Below, we provide a point-by-point response to all their comments, which have been (re-)numbered consecutively.
- recognizing work carried out for revision experiments (including protein purification and proteomics), we have added three authors to the manuscript.
- Although not explicitly requested, we have added a side-by-side (on the same plate) comparison of the inhibitory effects of Woldo and Danwoldo (part of new Figure S3, reproduced as Figure A below), which we thought would be useful.

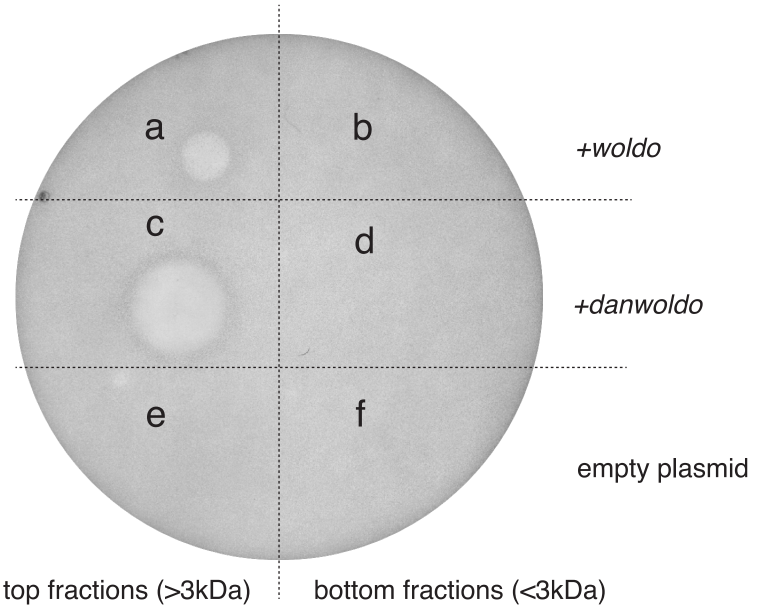

Figure A. Comparing the inhibitory effects of Woldo and Danwoldo on the same plate. The following supernatants were spotted onto a lawn of *H. halodurans,* a,c,e: top fraction (>3 kDa) of supernatant from *H. volcanii* (a) expressing Woldo, (c) expressing Danwoldo, (e) with an empty plasmid; b,d,f: bottom fraction (<3 kDa) of supernatant from *H. volcanii* (b) expressing Woldo, (d) expressing Danwoldo, (f) with an empty plasmid.

Reviewer #1:

General comments:

This is an exciting and important paper looking a much neglected field - natural antagonistic interactions between archaea and bacteria. The genomics-based observations are backed up by some proof-of-concept experiments in the lab, which are convincing. Nonetheless, I have some comments that should be considered.

Major comments:

1. *Phylogeny-based conclusions rely on decent MSAs so that the trees really represent protein evolution. MSAs should therefore be provided in supplementary materials.*

We agree with the reviewer. The alignments were already available on the project GitHub page mentioned in the manuscript (https://github.com/srom/archaea-vs-bacteria), but we had failed to explicitly highlight this. We now do. We have also elaborated the GitHub README file to facilitate re-use of the data.

2. *Additionally the scale of the trees is not clear: generally a substitution per site scale is provided, but what is the scale here?*

The tree scale provided is indeed substitutions per site. We now mention this in the legends to the relevant figures (Fig. 2, Fig. S1).

3. *2/3 of archaeal PGH homologs do not encode a known signal peptide. What do the authors think about potential function of these proteins?*

We find it difficult to meaningfully speculate about the function on these apparently signal peptide-less PGHs without knowing whether these proteins really do lack a signal for export or there *is* a signal but it is not recognized by current search methods, which are largely based on our knowledge of how secretion works in bacteria (and in a small slice of archaeal diversity). Our knowledge of signal peptides or similar secretion signals in archaea remains very much incomplete, especially in clades that are genetically inaccessible (e.g. Asgardarchaea), and we consider the data presented in Fig.1E as a lower-bound estimate of secretion. Assuming, for the sake of argument, that some of the archaeal PGHs are indeed not secreted but retained inside the cell, one might imagine that these could be involved in breaking down peptidoglycan imported into the cell (also see our response to comment #24). Alternatively, PGHs might be delivered to target cells by contractile injection systems, a possibility further discussed in our response to comment #11.

4. *The authors correctly point out that "pseudo-peptidoglycan (pseudomurein) exhibits different composition and linkage patterns, making it chemically distinct from bacterial peptidoglycan". However there are also many similarities, and some conclude that they have a shared ancestry (see doi: 10.1093/femsmc/xtab012). A simple BLASTP search would have established whether some of the species that have PGH homologs have a partial or complete pseudomurein biosynthesis cluster.*

We agree that it is interesting to ask whether archaeal PGHs might be acting on a peptidoglycan (PG)- or pseudomurein (PM)-like structure produced by their native organism.

Searching for genes that define the pseudomurein biosynthesis cluster is a reasonable strategy here. However, the full set of genes required for pseudomurein synthesis remains a subject of active research[1,2] and much of our knowledge derives from comparative analysis to the set of bacterial genes required for peptidoglycan synthesis. We therefore decided to tackle this question as follows:

First, we obtained HMM models for proteins encoded by the *division and cell wall* (dcw) gene cluster, which is well-conserved across bacteria, from the authors of a recent authoritative paper[3]. We then used these HMM models to survey all archaeal proteomes in our database (HMMER with e-value threshold of 1e-5, as in Ref. [3]) for the presence of dcw gene homologs.

The presence of any one of these genes in isolation is not indicative of the capacity to produce peptidoglycan or pseudomurein. MurA, for example, one of the genes in the dcw cluster, is common in Halobacteria whose cell wall architecture is well characterized and known to lack PG/PM.

We therefore asked which of the dcw proteins are found in *all* Methanobacteria and Methanopyri genomes in our database. These are the only two classes of archaea where pseudomurein production has been demonstrated experimentally. The closely related Methanococci archaea do not produce pseudomurein. We find only five genes to be universal to all Methanobacteria/Methanopyri but not to Methanococci or Halobacteria in our database: four muramyl ligase enzymes (*murCDEF*) and D-alanine-D-alanine ligase (*ddl).* These proteins have previously been highlighted as likely essential for PG/PM biosynthesis[1,2] and might – as highlighted by the reviewer – share a common ancestry between bacteria and archaea.

We then asked whether PGHs are more likely to be found in genomes that contain this "minimal essential set" for PG/PM biosynthesis. The answer is no. Of all archaeal genomes in our database, 353 (~10%) encode the minimal essential PG/PM set. Of these, 21 encode at least one PGH (6%). Of the 3,353 genomes that do not encode the full minimal set, 189 encode a PGH (5.6%). The relative incidence of PGH in genomes with and without the minimal set is not significantly different (Fisher test P=0.8). Perhaps more importantly, 189 out of 210 PGHs are found in genomes without the minimal set defined here where the archaeon is likely incapable of producing a PG/PM-like structure. While we cannot completely rule out that PGHs act on a PG/PM-like structure produced by their native organism, there is no support for this scenario based on our current knowledge of PG/PM synthesis and the distribution of PGHs across genomes with and without known PM synthesis.

It is also unlikely that the PGHs act on the PM of other archaea, given that the PM-producing clades inhabit a very different environment from most of the PGH-encoding archaea described here, including archaea from hypersaline environments like our experimental model *Halogranum salarium* (which – expectedly - does *not* encode the full minimal set of PM synthesis genes).

We have made the data and analysis results available via the project GitHub (https://github.com/srom/archaea-vs-bacteria) but we believe that it is not helpful – given the ongoing uncertainty about the genes involved in PM synthesis – to incorporate this analysis in the manuscript. Instead, we have highlighted the simple fact that only 2 of the 210 PGHs we describe come from known PM producers (Methanobacteria, Methanopyri).

Minor comments:

5. *The authors jump from phylum to order level in a way that is sometimes confusing, especially since some members of Halobacteriota are not halophiles.*

Deciding at which level of the taxonomic hierarchy to aggregate and present data is not always obvious. We found that, in our case, presenting data at the phylum level allowed us to illustrate relevant patterns across the archaeal tree while keeping visualizations informative. In some instances, however, we felt it better to focus on a lower taxonomic level (order or class, see Figure S1). In particular, we do this when the incidence of the focal protein is taxonomically restricted to that level and discussing incidence at a higher taxonomic level would leave the false impression that the gene is more widespread than it actually is.

6. *The choice of J2ZZK6 was probably also influenced by the fact that it has a secretion signal (TAT), this should be mentioned.*

Th choice of J2ZZK6 was indeed influenced by the fact that it bears a recognizable signal peptide. We now mention this in the first paragraph of the section "A peptidoglycan hydrolase from Halogranum salarium B-1 kills a halophilic bacterium".

7. *the archaeon can survive and grow at the lower salinities (up to 1.4M NaCl) - should be "down to" or "as low as"*

Corrected.

8. *the risk that media composition scuppers enzyme activity - to the best of my knowledge to scupper means to sink your own ship on purpose (metaphorically), I think "impairs" will be more accurate and much clearer to a non UK readership.*

We have replaced "scuppers" with "impairs".

9. *at fault for the bactericidal activity of the native supernatant. - should be "responsible for"*

Corrected.

10. *"We speculate that, for these species, PGH might nonetheless be useful when they encounter what, for them, are frontier environments: niches at the edge of their range, where lower salinity allows bacteria to be competitive and archaea survive rather than thrive. In the future, it will be interesting to define the (stress) conditions that trigger PGH expression and how they relate to the ecophysiology of the particular archaeon under investigation."*

    The authors seem to ignore the possibility of necrotrophy - because bacteria that are not high salt adapted may end up in evaporation pools, for example, where they will dehydrate and die, this could provide a source of nutrition for haloarchaea able to secrete PGHs.

In the original manuscript, we - very briefly - mentioned the possibility that bacteria might be food ("Or are bacteria themselves the resource, parcels of nutrition that are unlocked by PGHs?") rather than being killed because they are competitors. This is an interesting hypothesis, which can be tested in the future by monitoring, for example, archaeal growth rates in nutrient-depleted medium supplemented with dead bacteria (other things equal, archaea should grow better if they can use bacteria as a source of nutrition). Peptidoglycan hydrolases might indeed be used to unlock nutritional content, either through active killing (necrotrophy/predation) or by breaking down cells that are already dead (scavenging). Breaking down the cell wall of bacteria might be particularly useful in hypersaline environments: bacteria that die in this environment – following a salinity upshift or when bacteria enter this environment but are not adapted to it (e.g. bacteria that float in through the air and die on arrival) - do so by dehydration, as highlighted by the reviewer. Dehydration does not by itself compromise cell wall integrity, so organisms like archaea that might stand to benefit from dead cells will have to unlock the nutrients hidden within and PGHs would be a suitable tool for this purpose. Our work shows that exposure to PGHs can be lethal to *living* cells (suggesting competition or necrotrophy/predation) but does not rule out scavenging as a major use of PGHs. Establishing their primary adaptive value and understanding what drove acquisition and persistence of different PGHs will require substantial future work on the details of these interactions in ecological context.

We now elaborate more extensively on alternative (though not mutually exclusive) ecological scenarios in the Discussion.

11. Type VI secretion-based antagonism
- https://www.science.org/doi/10.1126/sciadv.adp7088 should be cited and referred to.

We now explicitly discuss type VI secretion systems (T6SSs) as an example of bacterial weaponry that is also in use by archaea in the section "A roadmap for the discovery of additional archaeal-bacterial interactions". Current evidence (e.g. the study by Zachs *et al.* cited above) supports use of these weapons against other archaea, and it will be interesting to establish whether bacteria are also targeted by archaeal T6SSs. We also highlight the observation that PGHs *without* a recognized signal peptide are enriched (P=0.059) in genomes that also encode phage sheath 1 protein (PF04984), indicative of the presence of a contractile injection system, which might be able to deliver the PGH to its intended bacterial target.

Reviewer #2:

The manuscript, titled "Systematic genome-guided discovery of antagonistic interactions between archaea and bacteria", systematically presents the distribution of peptidoglycan hydrolases in archaea. These hydrolases target the peptidoglycan in the bacterial cell wall and exhibit bactericidal activity. This research offers valuable perspectives on the potential of archaea-derived antibacterials and the relatively under-explored antagonistic interactions between archaea and bacteria within mixed microbial communities. I recommend the publication of this paper, following necessary revisions.

Main Comments:

12. *The study consistently focuses on peptidoglycan hydrolases through high-throughput genomic analysis and experimental validation. To more accurately encapsulate the essence of the research and distinguish it from other studies on antagonistic interactions, it would be beneficial to include "Peptidoglycan Hydrolases" in the title.*

Please see our response to reviewer comment #20.

13. *It is strongly recommended to clearly state the number of zoocin A homologs identified in archaeal genomes, as well as the distribution patterns of these analogs. Additionally, an explanation is needed regarding the rationale behind selecting only 10 of these homologs for the construction of the genetic tree analysis.*

We previously stated that we detect

> "homology against the peptidase M23 domain of zoocin A in **ten** [emphasis added] archaeal genomes in our database."

and that the

> "cell wall binding domain of *S. equi* zoocin A (zoocin A target recognition domain, PF16775) **is not found** [emphasis added] in archaea."

So the total number of archaeal modular PGHs with homology to zoocin A (via the M23 domain) is 10. [Note that we try to be careful here and speak of homology to a particular domain rather than "zoocin A homologs" because the native zoocin A domain architecture is not present in archaea.]

Of these 10 proteins, five are found in genomes belonging to the order Halobacteriales (as previously mentioned). While we provide a complete list of archaeal PGHs with domains homologous to M23 in Table S2, along with taxonomic information, we agree with the reviewer that we could have provided greater detail on this in the main text. We now do so, highlighting the observation that four of the five other M23 homologs are found in genomes from class Nanoarchaeia (with a single incidence in the phylum Thermoplasmatota).

It would be interesting to further characterize the PGHs from Nanoarchaeia and their potential contribution to cross-species interactions in the future, but none of the Nanoarchaeia genomes with PGHs are from species that have been cultured in isolation or are genetically accessible, so we naturally focused on Halobacteriales PGHs for in-depth analysis and downstream experimental work.

14. *In the manuscript, the names of the proteins should be presented in the results section rather than in the methods section. This would improve the logical flow and make it easier for readers to follow the key findings related to the proteins.*

We presume the reviewer means "discussion section" rather than "methods section" here and is referring to the names Woldo and Danwoldo. With hindsight, we agree with the reviewer

that it might be easier to follow the narrative with these given names instead of alphanumerical IDs and we have therefore implemented the change suggested by the reviewer, naming the genes/proteins upon first mention in the text. We also now use the given name throughout the figures.

15. Is it accurate that the RMSD value of the full-protein match is larger than that of the partial-protein match?

Yes, this is correct. Note that, in Figure 1B, which this question refers to, two *different* proteins are shown. On the left-hand side (full protein match), the bacterial PGH matches an archaeal homolog across almost all of its sequence with an RMSD of 1.29Å. On the right-hand side (partial protein match), the archaeal homolog matches the bacterial query PGH only partially, with each homolog exhibiting some structures that are not found in the other protein. Importantly, the quality of the match is computed only over the matching region (RMSD = 3.06Å). The labels full or partial protein match don't tell us how good the structural homology is in the part of the protein that does match. There is no expectation that RMSD should be higher or lower for a full or partial match, as RMSD is computed only across the alignable part of the two structures that are being compared.

16. The protein-domain-level genetic tree of peptidase M23 reveals an unusual phenomenon: proteins from haloarchaea with similar domain structures are not clustered together. This unexpected result requires in-depth analysis and a more detailed explanation.

The domain-level trees presented in Figure 2 do indeed suggest a complex evolutionary history. The tree for PG binding 1 domain appears the more straightforward to interpret: Archaeal PG binding 1 domains are monophyletic and, within this archaeal group, the branching topology is broadly consistent with the species topology (making some allowance for nodes with low bootstrap support). This is consistent with a single ancestral acquisition and subsequent maintenance (and loss in lineages that are not represented here). At the same time, it is already evident from this tree that the catalytic partner domain to PG binding 1 has been swapped a number of times so that we find PG binding paired with amidase 2, peptidase M23, and glucosaminidase domains in different archaea. The same is true in bacteria displayed in the same tree. This domain modularity has previously been highlighted as a general property of peptidoglycan hydrolases[4]. Some of the catalytic domains might have been acquired (and then fused with PG binding 1) via horizontal transfer. In this context, it is perhaps a bit easier to rationalize the pattern of evolution shown in Figure 2A, for the M23 domain, which is consistent with a model where the M23 domains in archaeal PGHs were acquired twice from different donor bacteria, and are therefore not monophyletic. Given the paucity of sequences, there is not a whole lot of additional formal analysis we can do to further support this scenario, but we now elaborate on this origin scenario more extensively when discussing Figure 2.

17. While the in-vitro hydrolysis validation experiment was performed using the crude extract of the heterologous expression and the supernatant of the wild-type strains, a more definitive conclusion could be drawn by repeating the experiment using pure protein and substrates, which is feasible.

We agree that it would be nice, ultimately, to demonstrate peptidoglycan hydrolysis using purified proteins and substrates. As part of this revision, we have made a first important step towards this goal. Using an AKTA protein purification system, we fractionated supernatant from Danwoldo-expressing *H. volcanii* strains and then tested individual fractions for activity on *H. halodurans* lawns. The top left panel of Figure B below shows the killing fractions. While a protein of the expected size (see arrow) is present in the killing fractions (Figure B, right panel) and absent from the empty plasmid control, the fractions also contain some other proteins. We therefore subjected killing (and neighbouring) fractions to quantitative mass spec analysis, which a) confirmed that the most prominent band in the killing fractions is indeed Danwoldo, and b) shows that killing activity tracks Danwoldo abundance (Figure B, bottom panel). Other prominent bands in the gel correspond to well-known members of the Haloferax cell surface (pilin A, coxB1, petA) that are not expected to be involved in killing activity, but likely found in these fractions because they are so abundant that degradation fragments are very common. We now describe these experiments in the revised manuscript text and include Figure B as part of the new Figure S3.

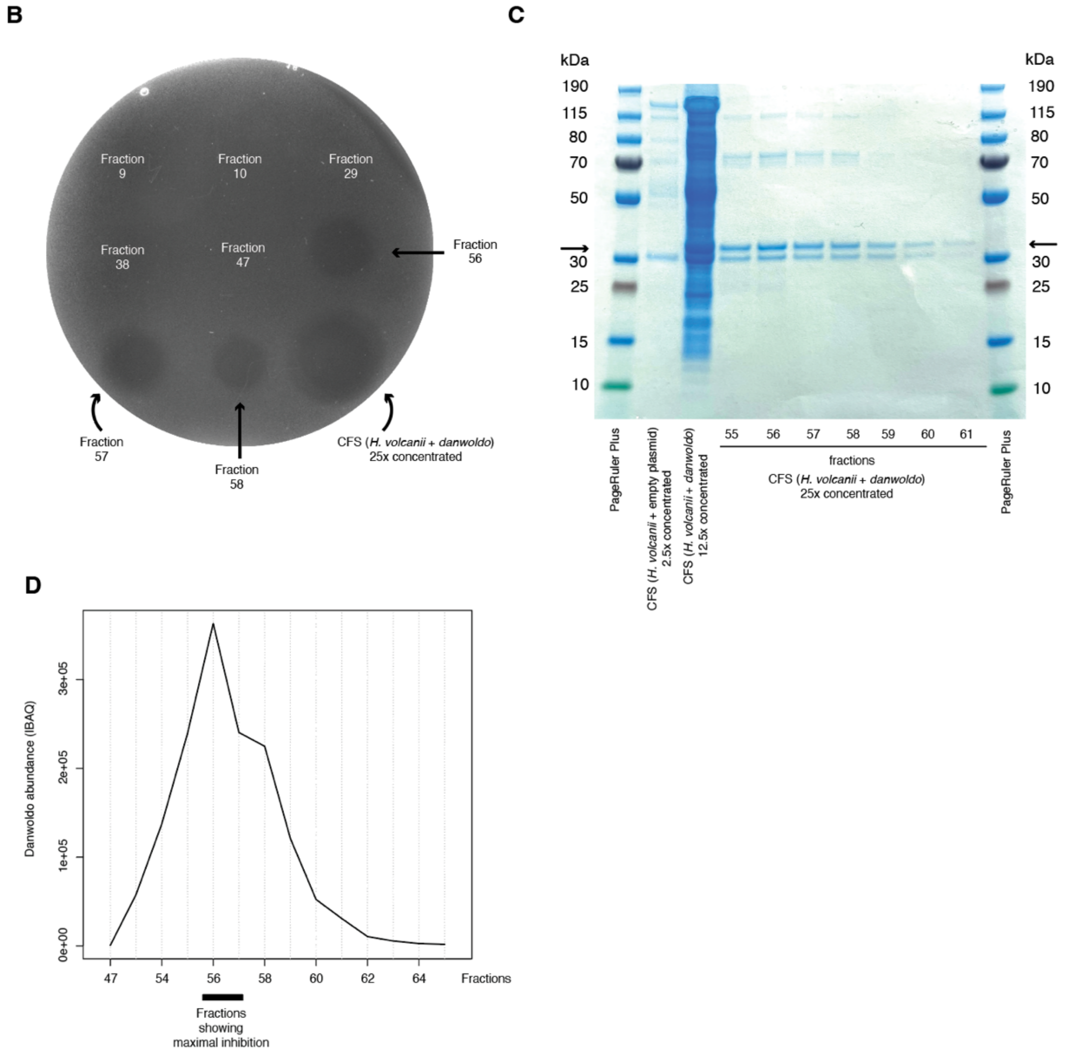

Figure B

18. Considering that the subsequent protease J2ZJU5 can be predicted in silico and is forecasted to possess a signal peptide, the author should provide a rationale for the continued use of proteomics analysis to guide the discovery process.

The reviewer is right that we can (and did) predict both J2ZZK6 and J2ZJU5 as PGHs *in silico*. The rationale for carrying out proteomics was therefore not to guide discovery but to understand whether these proteins are not just present in the genome but actively secreted and could therefore account for bactericidal activity of the native supernatant. Presence in the genome sequence does, after all, not guarantee expression of the protein. We have rephrased this paragraph to clarify the rationale for carrying out supernatant proteomics.

19. Figure 4, as it stands, appears to be disconnected from the overall logical structure of the manuscript. Its relevance should be reassessed, or alternatively, a clear link to the central argument should be established within the discussion section.

Although we agree that Figure 4 is not tightly related to the PGH-centric narrative of the majority of the manuscript, we nonetheless think it is important because it suggests that archaeal-bacterial conflict goes beyond PGHs, providing important motivation for future work. We would therefore like to retain this figure and believe it is appropriately contextualized.

Reviewer #3

This interesting manuscript reports the identification of peptidoglycan hydrolase genes in archaeal genomes and that the corresponding enzymes are presumably used to target bacteria in interspecies competition. The supplemental files with the genes/proteins identified will be a valuable source for the field. Unfortunately, the manuscript has not page/line numbers.

Main points:

20. I suggest changing the title, which claims that they identified antagonistic interactions between archaea and bacteria. However, this has not been tested. To test for antagonistic interactions, they would have to co-culture a target bacterium with a hydrolase-producing archaea in a competition assay that quantifies survival of both. At present, they can only conclude that some archaea secrete peptidoglycan hydrolases and two of these can kill a bacterium that might occupy the same environmental niche. In my view this is already an important result.

We appreciate the reviewer's point here. In response, and because we thought that this would add a valuable new dimension to our work, we have tried over the last few weeks to establish co-cultures between *H. salarium* and *H. halodurans* to see if we can demonstrate antagonistic interactions directly. Unfortunately, in the time we allowed ourselves to complete these revisions, we have not managed to do so. The main stumbling block is that the salt concentration ranges at which these two microbes grow barely overlap. While *H. halodurans* grows ok at 8% w/v NaCl, it does not grow beyond 10% whereas *H. salarium* grows optimally at ~15% and barely grows at 8%. The end result is that both organisms grow poorly

in the overlapping range of concentrations, and if we shift salt concentrations either up or down, one of the two fails to establish itself or to reach appreciable densities in co-culture conditions. We have experimented with salt gradients to mimic a natural transition zone, which would allow both to become established yet be exposed to each other's secretomes, but this set-up will require further optimization to obtain robust results, taking this outside the scope of this study (but very much inside the scope of our future efforts in this area).

Given the lack of success in getting these co-cultures off the ground, we are happy to change the title. Also taking into account the request by reviewer #2 (comment #12), we suggest the following simple but informative title: "*Archaea produce peptidoglycan hydrolases that kill bacteria*". However, we are open to alternative (editorial) suggestions.

21. They searched for archaeal genes that encode for a peptidoglycan hydrolase domain and a peptidoglycan-binding domain. This combination of an enzymatic and a binding domain is mainly found in bacterial enzymes that target Gram-positive bacteria. There are many peptidoglycan hydrolases that lack a peptidoglycan binding-domain and target Gram-negative bacteria or even Gram-positives. A well-known example is lysozyme. Hence, they should clearly state that their strategy does not identify single-(enzymatic) domain hydrolases and could be biased towards hydrolases that target Gram-positive bacteria.

We thank the reviewer for highlighting this. We agree and now state this limitation prominently in the text (first paragraph of the Results). Of course, there is a very good reason that we focus on PGHs with a modular (catalytic+binding domain) architecture: many of the catalytic domains, when found as standalone domains, can act on a number of different substrates. For example, amidase domains can be involved in cleaving peptide bonds in multiple contexts that have nothing to do with peptidoglycan (e.g. acting during protein degradation). By focusing on PGHs with modular architectures, we trade off sensitivity (spanning more potential cases of archaeal-produced PGHs) for specificity (increasing the chance that the proteins we identify do indeed act on peptidoglycan), which we believe is a worthwhile trade-off here.

22. The introduction lacks the mentioning of peptidoglycan hydrolases secreted by type VI systems and targeting Gram-negatives, which shows again a bias towards Gram-positives in the approach.

We now discuss type VI secretion systems in greater depth ("A roadmap for the discovery of additional archaeal-bacterial interactions"), including the possibility that they might deliver catalytic-domain-only PGHs into gram-negative bacteria (see our response to comment #11).

23. They found that many archaeal hydrolase genes are present on plasmids. Can they provide evidence that the hosts of these plasmids are archaea and not bacteria living in the same environment?

The plasmids we mention are exclusively archaeal plasmids, at least in the sense that they were assigned to specific archaeal strains/genomes. These assignments were not done by us; we simply inherit them from upstream genome assembly and annotation efforts which end up

being deposited in NCBI. How assignments were initially made will vary depending on the study and genome submitter. In the easiest case, plasmids are from strains grown in isolation and therefore trivial to assign. In other cases, e.g. where we are dealing with metagenome-assembled genomes (MAGs), assignments can rely on a variety of genomic features, including the presence of specific transfer or replication systems, homology to a main chromosome, or the presence of CRISPR spacers (see Ref. [5] for a nice recent summary). This depends on the particular authors. We do not see a reason to distrust those assignments.

24. *They should more clearly discuss that not all secreted peptidoglycan hydrolases might have a role in killing bacteria. Archaea might secrete hydrolases to digest the cell wall of lysed bacteria present in the environment, to access the nutrients. Did they check whether archaea that contain peptidoglycan hydrolases have genes encoding peptidoglycan uptake and processing factors?*

To address this point, as we did in response to comment #4, we carried out homology surveys (with phmmer) for known peptidoglycan uptake factors, taking as our starting point a list of peptidoglycan transporters summarized in a recent review on the subject[6]: OppBCDF-MppA, AmpG, YejBEF-YepA, CadB, NagE, MurP, and MurT. 963 out of 3,706 archaeal genomes (26%), so a fair number, encode one or more of these systems. Looking across these systems, genomes with a PGH (57/963=5.9%) are as likely as genomes without a PGH (118/2743=4.3%, Fisher test P>0.05) to encode one of these systems. We then asked whether genomes with a modular PGH were enriched for any one particular system compared to genomes without PGHs. Interestingly, we find that genomes with a PGH are more likely to encode OppBCDF-MppA (40 out of 175, ~23%) than genomes without a PGH (298 out of 3531, ~8%, Fisher test P=1.76e-8). There was no enrichment/depletion for any of the other transport systems. *H. salarium* B-1 is one of the genomes encoding OppBCDF-MppA. It is interesting to speculate that organisms whose genome encodes both a PGH and OppBCDF-MppA might using peptidoglycan as a food source and we now mention this possibility in the Discussion.

25. *They identified two peptidoglycan hydrolases in Halogramum solaris that seem to kill a halophilic bacterium. Can they add a scheme to indicate the genomic context of these hydrolase genes? What other genes are in their neighbourhood and are they part of an operon?*

We agree with the reviewer that it is interesting to examine genomic context. Functional genes often cluster in prokaryotic genomes and one can therefore, on occasion, learn more about the function of a focal gene by considering its neighbours. The genomic context of *woldo* and *danwoldo* in *H. salarium* B-1 are reproduced below as Figure C and have been added to the manuscript as new Fig. 2D). Note that the coordinates look close but the two genes are actually on two different contigs. *Woldo* is predicted (by Operon-mapper) to be in an operon with an upstream gene of unknown function.

One potentially interesting observation is that both *woldo* and *danwoldo* have a number of (different) hydrolases in their neighbourhood, including an alpha amylase, a glycoside hydrolase, and alpha/beta hydrolases, some or all of which might be involved in carbohydrate processing. Whether these enzymes act (or are expressed) in concert with Woldo/Danwoldo and perhaps form part of a joint response (e.g. to poor nutritional status) remains to be established. At this point, we don't think the observation is particularly compelling, in part because there is no apparent conservation of synteny/genomic context/operon membership

with the Haladaptus genomes that encode *woldo/danwoldo* homologs (illustrated in Figure D below using *Haladaptus cibarius* as an example).

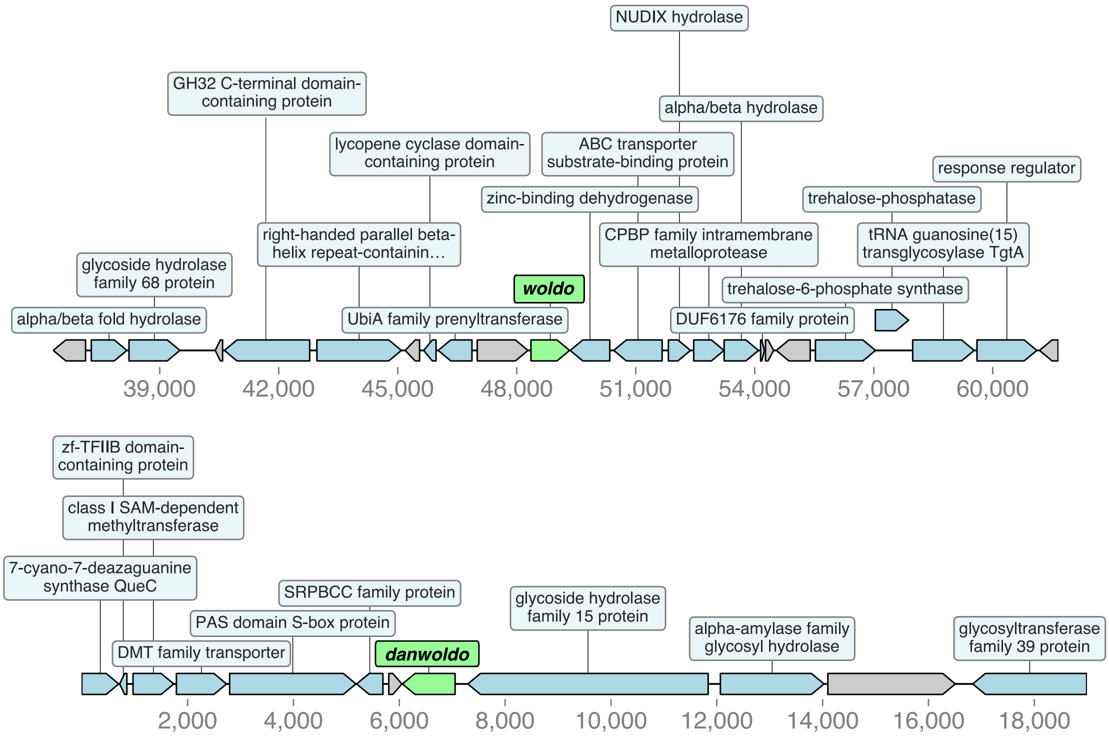

Figure C. Genomic context of *woldo* and *danwoldo* in *H. salarium* B-1. Genes coloured grey are hypothetical.

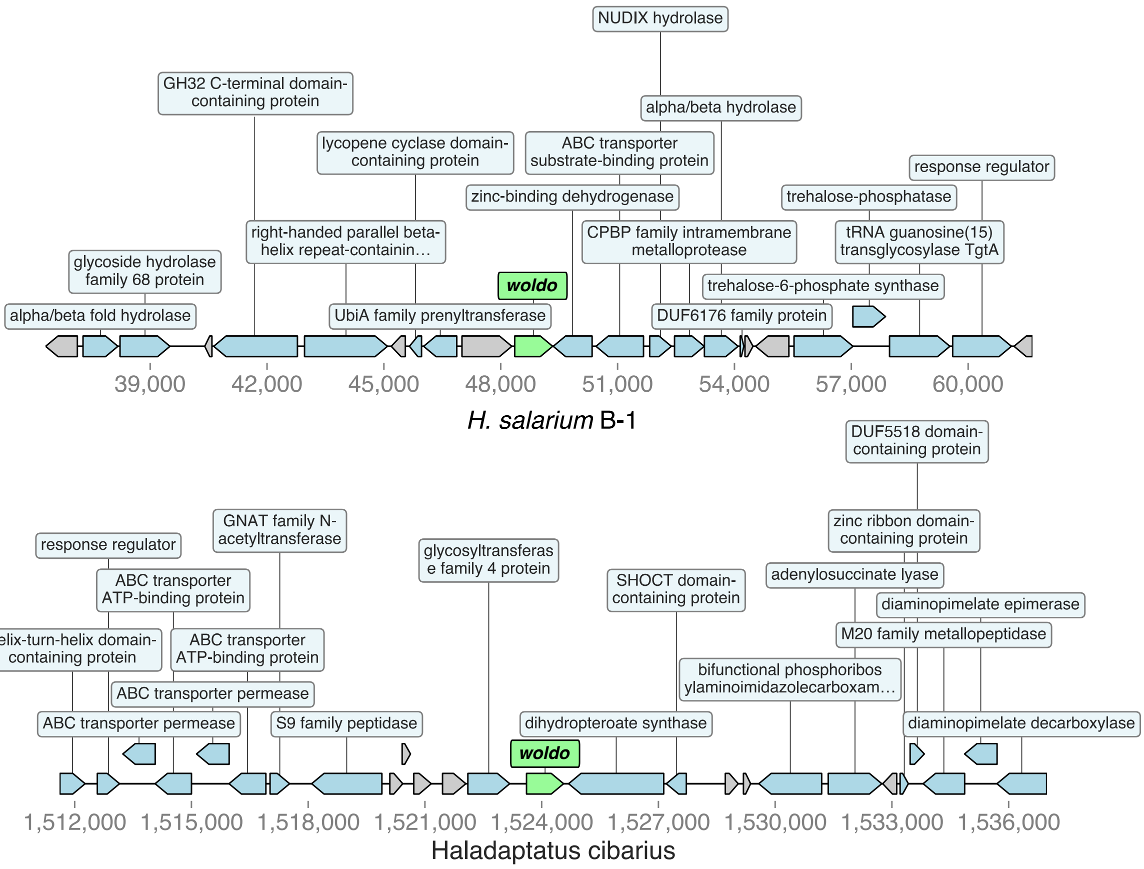

Figure D. Genomic context of *woldo* in *H. salarium* B-1 and *Haladaptatus cibarius.* Genes coloured grey are hypothetical.

26. Fig. 3G: should the axis label be colony forming units per ml? Can they explain the error bars and number of repeats in the figure legend?

The dilution factor was the same for all conditions so the graph looks the same but we agree with the reviewer that this is best expressed in CFU/ml and have amended the figure accordingly. Error bars are now defined and the number of repeats indicated in the figure legend.

27. Fig. 3H: the size bars are barely visible in the images and not explained in the figure legend.

Our apologies. The scale bars were indeed barely (no pun intended) visible. We have made them thicker.

Reviewer #4

This manuscript by Strock et al., which I'm assuming I received after the first round of revisions, presents a deep exploration of peptidoglycan hydrolases in Archaea as potential

mediators of antagonistic interactions. They undertook a well thought-out integrative strategy, from phylogenetic analyses all the way to mass spectrometry and classic microbiological techniques when determining the activity of the J2ZZK6 peptidase. I wish to especially commend the authors on their stylistic approach and their essayist structuring and language, which I feel we ought to see more in manuscripts. Save for a few minor clarifications, I would strongly recommend that the manuscript be accepted almost as is.

1) Results

28. "phylogenetically balanced database": Where any genome quality criteria applied in the choice of genomes?

Yes, quality criteria were applied, although indirectly. As described in the Methods section, starting from the complete GTDB database, we select

> "a single representative strain per species (as per the representative strain flag available in GTDB) and a maximum of ten species per genus, prioritizing those species with most strains"

Selection of the representative strain by GTDB prioritizes genomes assembled from the type strain of the species, but also considers the overall quality of the assembly[7]. So yes, there are explicit quality criteria applied by GTDB to designate representative strains and we make use of these criteria by choosing to focus on those representative strains.

We have added an explanatory note to the methods section to highlight this point.

29. Did the process of assembling the set of genomes result in certain environments being undersampled? Moreso for Bacteria; for Archaea the only undersampled taxon case I might expect are some genera in Methanobacteriales (maybe Methanosarcina or certain Halos?), though I would need to check the numbers for GTDB r214.

The sampling is designed to capture phylogenetic rather than environmental diversity. Here, we capture diversity fully at the genus level and include up to ten species per genus (see Methods). So biases, if they exist, will only apply to genera with >10 species, where not all genomes are included. In these cases, we select genomes based on non-environmental criteria (via pre-existing GTDB flags, see our response to comment #28). It is possible that this inadvertently introduces some environmental biases reflective of biased sampling strategies in the microbiology community (e.g. the diversity of human host-associated strains of a given genus is probably higher than its representation across all biomes), but we expect such biases to be subtle.

30. "superphyla": There are no superphyla in the GTDB classification as of r220. Previously dubbed superphyla have been downgraded to phylum level (e.g., TACK to Thermoproteota, all Asgards to Asgardarchaeota). Iirc, only DPANN could be informally

deemed a broadly accepted superphylum (and maybe Methanomada: Methanobacteriota+Methanobacteriota_A).

We thank the reviewer for this correction. We have removed the outdated mention of superphyla and now simply say that PGHs are found in many "archaeal genomes, including representatives from most archaeal phyla".

31. "we find M23 C-terminal of a LysM domain": in the C-terminal direction/side of ... ?

We have rephrased this sentence to clarify.

2) Discussion

32. J2ZZK6: If the protein is zoocin-related, do any of the potential targets have a zoocin immunity factor or any identifiable putative defense systems?

This is an interesting question. First, there are no "narrow-sense" (blastp, default settings) homologs to the zoocin A immunity factor (zif) in either of the three bacteria we tested (*H. halodurans, P. endophyticus, V. salexigens*). Looking more broadly for proteins that contain the same domain as Zif (Pfam: FemAB, PF02388; InterPro FemABX, IPR003447), we do retrieve hits in all these genomes, but they are annotated as lipid II:glycine glycyltransferases, indicating that the FemABX domain is not necessarily acting on peptidoglycan. In line with this, the domain is quite common in both bacteria and archaea. So, at present, we don't think the presence of this domain per se can tell us whether peptidoglycan has been modified to resist zoocin A-like cleavage.

33. Another point is structure of J2ZZK6: the Alphafold prediction includes an intrinsically disordered N-terminal region. Most of that segment registers as NlpD on CDD but the sequence and disorder vaguely reminded of a Tat signal peptide. Tat signal peptides are found in halocins (https://doi.org/10.3389/fmicb.2012.00207) and afaik are common in Halobacteriales. A quick BLAST search shows no homologous segments in many Bacteria. I believe that a useful analysis for future work would be to classify the signal peptides of peptidoglycan hydrolases in Archaea and whether they were added, switched, or lost following the HGT event.

We had already systematically predicted signal peptides for all archaeal PGHs (see Figure 1E and Table S2). Both J2ZZK6 (Woldo) and J2ZJU5 (Danwoldo) are predicted to have a Tat signal. We now explicitly highlight this in the text (see response to comment #6). It would indeed be interesting to know how these TAT signals were gained during evolution, not only for PGHs but for any protein that is gained via HGT and ends up being secreted. We note that, while Tat signals are rarer than Sec signals, Tat signals do exist in bacteria. So one possibility is that a PGH arrived with a Tat signal, ready for export from the archaeal cell, but the signal peptide (along with the remainder of the sequence) subsequently diverged to lose strong traces of its bacterial ancestry.

34. "a valuable tool for identifying producer-target relationships": While I do agree with this statement, to what extent can the workflow be generalized to other peptidoglycan hydrolases? For example, do the corresponding databases and tools already exist?

In principle, the workflow we present can be generalized to any protein that has a cell wall binding domain. This includes proteins that do not encode domains for catalytic/hydrolytic activity. In fact, there are quite a few PG binding domains in archaea, especially nanoarchaea, that are not coupled to catalytic domains, and which might be used to establish (perhaps commensal) interactions with bacteria inhabit the same niche. As we focused on antagonistic interactions here, we did not explore this further. Unfortunately, we are not sure what the reviewer means when they ask whether "the corresponding databases and tools already exist".

35. *"act as keystone species": When mentioning keystone species, the (Moissl-Eichinger et al., 2018) review references (Tajima et al., 2001) and (Tapio et al., 2017), both of which discuss methanogens. Halos might be keystone species in their respective environments although I'm not readily cognizant of any literature on the subject. Generalizing to all Archaea without an analysis of the ecological distributions of PGHs seems like overreaching.*

We have amended this section to read "Because of their unique metabolic capacities, at least some archaea likely act as keystone species". We hope this phrasing appropriately acknowledges our limited knowledge here.

36. *"are frontier environments": This is fairly reasonable speculation but I would suggest a couple of additional bioinformatic analyses that could support it pre-emptively. First, when looking for potential PGH targets, for each GTDB species cluster, find the archaeal MAGs. Go to the corresponding SRA/BioProject and constrain the search to only bacterial MAGs therein. Other species in the cluster might also originate from frontier environments. Second, I would hypothesize that the halophily amino acid composition bias (D+E/I+K in https://doi.org/10.1038/s41564-024-01647-4) and/or the isoelectric point (https://doi.org/10.1016/j.mib.2015.05.009) of PGHs deviates from other secreted proteins, if they are active in frontier environments.*

Regarding the first suggested analysis, unfortunately, we don't really get what the reviewer suggests we do. The second suggested analysis, we do understand. To look at this, we calculated the pI of haloarchaeal PGHs and compared it to the pI of all other secreted proteins (as adjudged from the presence of a signal peptide) from the same genomes (i.e. only those genomes that encode a PGH so that the proteins being compared evolved in the exact same environment). The results are shown in Figure E below. There is a slight tendency for the pI of PGHs to be higher than that of other secreted proteins from the same genomes. Although this is nominally supportive of PGH activity in less salty (more frontier) environments (lower pI being an adaptation to higher salt), and therefore consistent with our tentative hypothesis, we are reluctant to claim this in support. There are a number of reasons (perhaps simply to do with constraints on amino acid composition) why PGHs might deviate from other proteins and whether PGHs really are deployed in frontier settings is a question that will ultimately require in vivo experiments and/or co-culture observations under ecologically relevant conditions.

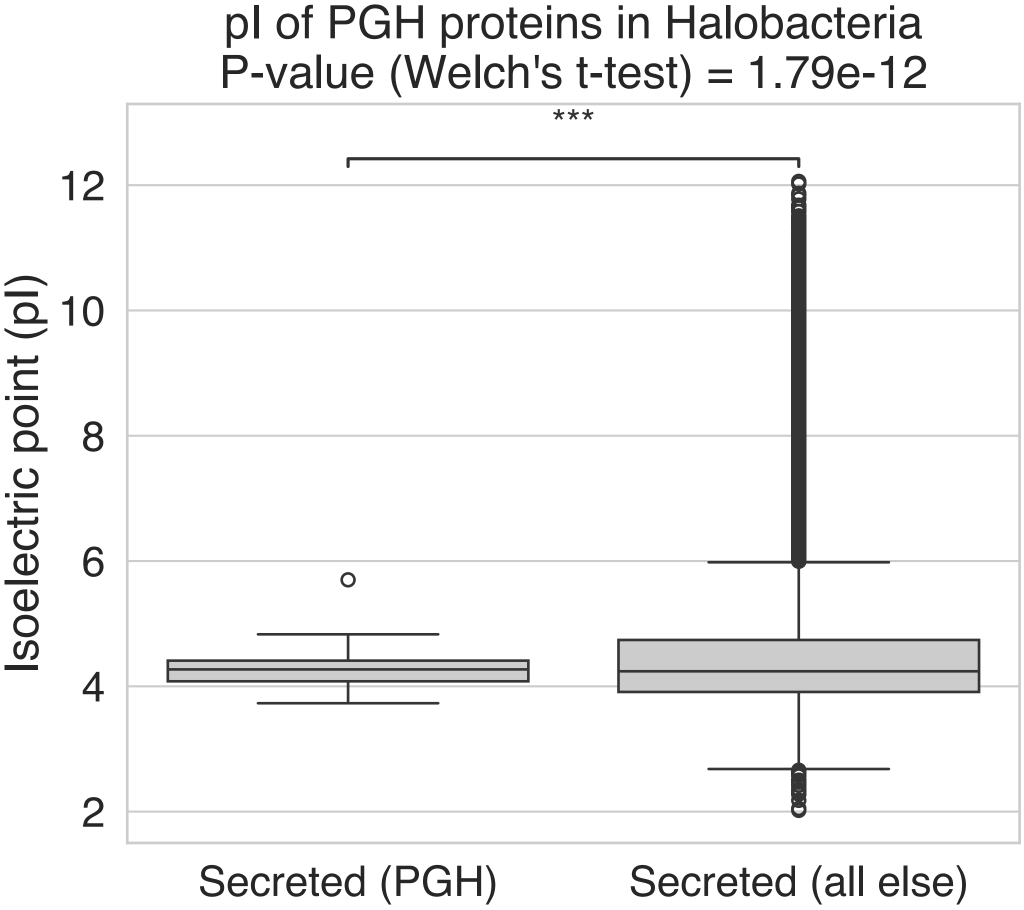

Figure E.

3) Methods

37. "all named phyla were kept": Why? There is a wealth of genomes in certain "alphanumeric" phyla, for exampled SpSt-1190 and B1Sed10-29 in the DPANN.

This was done simply for visualization purposes (Figures 1C and 4). The homology searches included all species in our database, irrespective of whether phyla were named or not, and the resulting hits (including a hit against the B1Sed10-29 phylum, mentioned by the referee) are available in Table S2. We have clarified this in the relevant part of the Methods section.

38. "less than 35% aligned residues": What was the rationale for the choice of percentage? Something in the literature or otherwise? I'm personally a fan of the smart-gap trimming mode in ClipKIT.

The cut-off was motivated by recent work on structural phylogenomics, which used <35% aligned residues as their cut-off for alignment trimming[8]. We have added a reference to this work in the relevant part of the Methods section. We chose to adopt this threshold in case we ended up exploring structural alignments (which, in the end, we did not). Of course, any hard cut-off is somewhat arbitrary, and we thank the referee for highlighting the smart-gap trimming mode of ClipKIT, with which we will familiarize ourselves for future work.

39. "bacterium's NCBI taxonomy ID": Did you encounter any issues, due to GTDB-NCBI taxonomic discrepancies?

For this analysis, we are matching a bacterial genome from the virus-host database (which provides NCBI taxonomic IDs) to our subset of the GTDB database. A given NCBI taxonomic ID can point to >1 GTDB genome. Whenever this was the case here, we picked the GTDB genus representative genome ("gtdb_type_species_of_genus" in GTDB) if available or else the genome with the highest completeness ("checkm_completeness" in GTDB). We have added this additional explanation to the relevant part of the Methods section.

40. "target proteins using ColabFold": How many proteins were in that set? Was ColabFold run locally?

There were 1,372 proteins in that set (as documented on GitHub and now also mentioned in the revised Methods text). ColabFold (v1.5.2) was installed using EasyBuild on Imperial College's High Performance Computing cluster (with CUDA library 11.7.0 for GPU acceleration on Nvidia A100).

41. "primary sequence or structural homology": In general, "homology" is not quantitative, so you would need to rephrase to "sequence identity" (did you mean "primary structure" or "primary and tertiary structure"?) and some equivalent for structures e.g., RMSD.

This has been correct to read "...target proteins were ranked either by protein sequence identity or structural similarity (measured as RMSD) to the archaeal query domain...".

42. Due to the broad interest of the topic for ecologists, evolutionary, and structural biologists, I hope that the authors will continue to further refine their approach. I would be interested in seeing more archaeal peptidoglycan hydrolases characterized and their structures solved, from environments where Archaea abound; from the gut microbiome to environments under severe energetic constraints, such as the deep subsurface.

We would love to see more archaeal PGHs tested for bactericidal activity and characterized in greater detail. We hope this work encourages others to join us in this endeavour, with a longer-term objective of understanding archaeal-bacterial conflict across the full breadth of environments that archaea inhabit.

References

1. Subedi, B. P., Martin, W. F., Carbone, V., Duin, E. C., Cronin, B., Sauter, J., Schofield, L. R., Sutherland-Smith, A. J. & Ronimus, R. S. Archaeal pseudomurein and bacterial murein cell wall biosynthesis share a common evolutionary ancestry. *FEMS Microbes* **2,** xtab012 (2021).

2. Lupo, V., Roomans, C., Royen, E., Ongena, L., Jacquemin, O., Mullender, C., Kerff, F. & Baurain, D. Identification and characterization of archaeal pseudomurein biosynthesis genes through pangenomics. *mSystems* **10,** e01401-24 (2025).

3. Megrian, D., Taib, N., Jaffe, A. L., Banfield, J. F. & Gribaldo, S. Ancient origin and constrained evolution of the division and cell wall gene cluster in Bacteria. *Nat. Microbiol.* **7,** 2114–2127 (2022).

4. Baba, T. & Schneewind, O. Target cell specificity of a bacteriocin molecule: a C-terminal signal directs lysostaphin to the cell wall of Staphylococcus aureus. *EMBO J.* **15,** 4789–97 (1996).

5. Maddamsetti, R., Wilson, M. L., Son, H.-I., Zhou, Z., Lu, J. & You, L. Scaling laws of plasmids across the microbial tree of life. *bioRxiv* 2024.10.04.616653 (2024). doi:10.1101/2024.10.04.616653

6. Gilmore, M. C. & Cava, F. Bacterial peptidoglycan recycling. *Trends Microbiol.* **33,** 340–353 (2025).

7. Parks, D. H., Chuvochina, M., Chaumeil, P.-A., Rinke, C., Mussig, A. J. & Hugenholtz, P. A complete domain-to-species taxonomy for Bacteria and Archaea. *Nat. Biotechnol.* **38,** 1079–1086 (2020).

8. Puente-Lelievre, C., Malik, A. J., Douglas, J., Ascher, D., Baker, M., Allison, J., Poole, A., Lundin, D., Fullmer, M., Bouckert, R., Kim, H., Steinegger, M. & Matzke, N. Tertiary-interaction characters enable fast, model-based structural phylogenetics beyond the twilight zone. *bioRxiv* 2023.12.12.571181 (2024). doi:10.1101/2023.12.12.571181
